# Multidisciplinary management and care in rare neuromuscular disorders: A call for action

**DOI:** 10.1111/ene.16265

**Published:** 2024-02-29

**Authors:** Kristin Ørstavik, Andreas Dybesland Rosenberger, Hanne Ludt Fossmo, Marianne Nordstrøm, Marianne de Visser

**Affiliations:** ^1^ Unit for Inborn and Hereditary Neuromuscular Disorders, Department of Neurology Oslo University Hospital Oslo Norway; ^2^ National Neuromuscular Centre Norway University Hospital of North Norway Tromsø Norway; ^3^ Vikersund Rehabilitation Centre Vikersund Norway; ^4^ Frambu Resource Centre for Rare Disorders Siggerud Norway; ^5^ Department of Neurology, Amsterdam University Medical Centre Location University of Amsterdam Amsterdam The Netherlands

Rare neuromuscular disorders (NMDs) comprise a large spectrum of diseases that may be present at all ages, at birth or they may develop during early infancy or childhood, in adulthood, or late in life. The European definition of a rare disease is one that affects <1 in 2000 people. Many are hereditary, such as spinal muscular atrophy (SMA), Duchenne muscular dystrophy (DMD), myotonic dystrophy, and Charcot‐Marie‐Tooth disease. Others are acquired, such as amyotrophic lateral sclerosis (ALS) and inflammatory myopathies, are important differential diagnoses in neurological practice.

Common for the rare hereditary NMDs is that they often affect several family members, most progress throughout life, and they are usually associated with a severe disease burden. In a fair proportion, the heart and respiratory function are also involved, as well as the ability to chew and swallow. For the majority of these disorders, we have thus far been unable to provide any targeted medical treatments. Nevertheless, international care recommendations have been developed for several of the most serious disorders, acknowledging the involvement of several organs and functions [1, 2, 3]. These recommendations advocate a multidisciplinary approach that integrates various medical specialties and health care professionals. Multidisciplinary care has been associated with better adherence to clinical follow‐up, resource utilization, and quality of life [4]. However, there is limited knowledge regarding the compliance with these recommendations, and when studied, such as in DMD, there are many barriers to successful practice [5]. To our knowledge, there is inadequate use of multidisciplinary teams and sparse research on the topic. This is especially true for the adult population with hereditary NMDs. In contrast, there are several studies on the positive effects of a multidisciplinary approach in the acquired NMDs, especially ALS [6]. Paganoni and coauthors published a review in 2017 confirming that a multidisciplinary approach is recommended in rare neuromuscular disorders, but that there is a need for research on quality and cost‐effectiveness of multidisciplinary clinics [4]. They point out that there is an urgent need to show that multidisciplinary teams are worthwhile.

Recently, we took the initiative to establish a group for multidisciplinary management and care in the European Reference Network Euro‐NMD, covering rare neuromuscular disorders. In the first hybrid workshop held in Norway in May 2023, representatives from a patient advocate group as well as health professionals from eight different European countries participated. During informal discussions and lectures, it became obvious that many of these patient groups do not have access to multidisciplinary teams. This calls for action, and we are of the opinion that as new orphan drugs are being developed, the need for multidisciplinary follow‐up and care will become even more apparent. Overall, given that recommendations are established to facilitate the attainment of best practice, it is imperative for health authorities to prioritize multidisciplinary teams. Figure 1 presents a model of specialized health care services organized around a multidisciplinary core team with expertise in diagnostics, treatment, and disease monitoring. Furthermore, these core teams require allocated resources to effectively coordinate care, and when necessary, foster interdisciplinary collaboration with other health care professionals and hospital departments.

**FIGURE 1:**
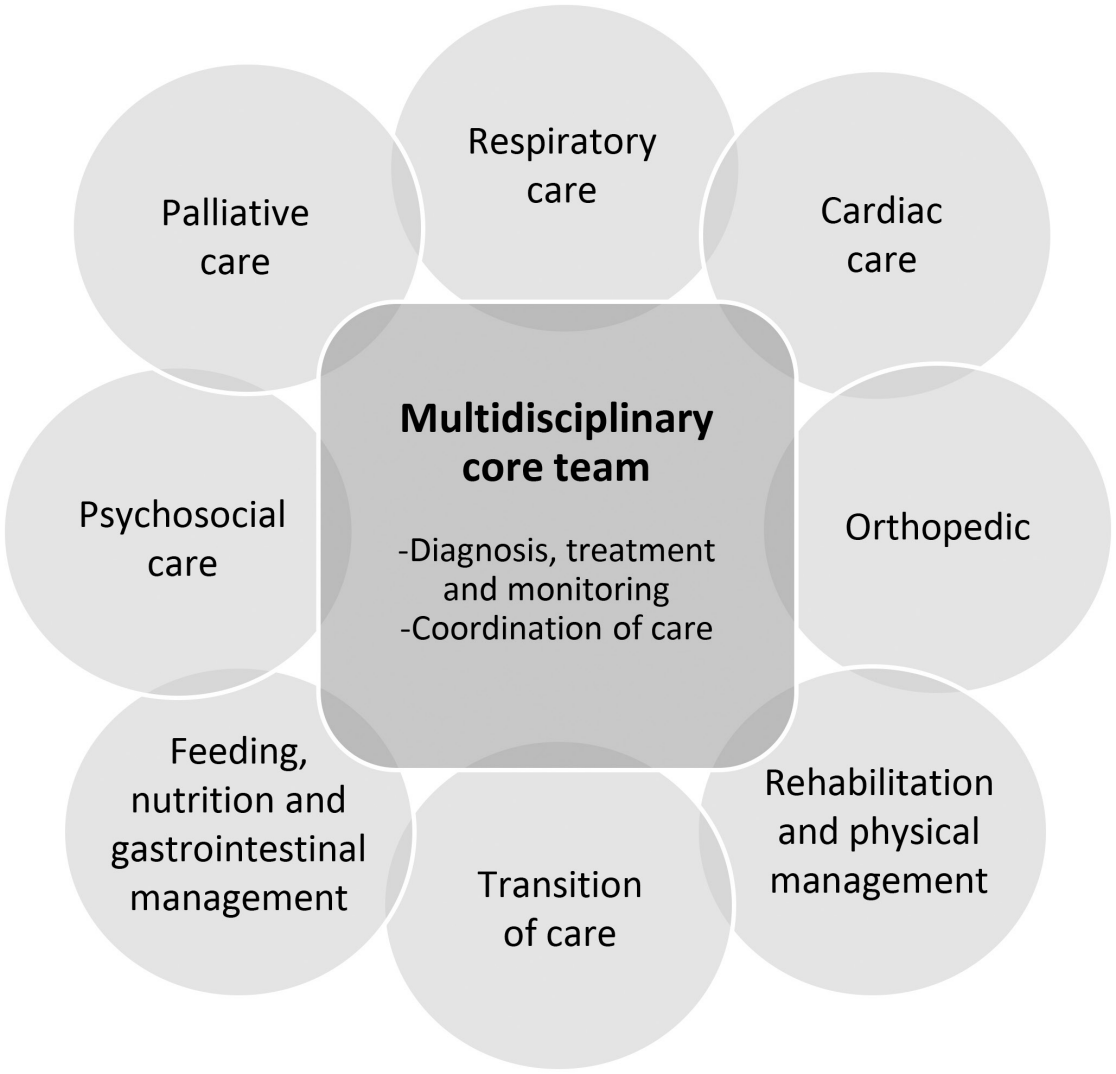
Model of multidisciplinary specialized health care services in hereditary neuromuscular disorders with examples of interdisciplinary care areas.

The revolutionary transformation in the treatment approach for children with SMA serves as an example. SMA is an autosomal recessive hereditary disorder that affects the lower motor neurons due to the lack of survival motor neuron protein (SMN). There are four different types of SMA dependent on when the symptoms appear, and what motor milestones the child reaches. Approximately 50% of the children with SMA develop the most serious type, SMA 1, and if untreated, they usually die before the age of 2 years. Over the past years, three different targeted treatments have become available for children with this disorder, including gene therapy. This has led to many countries adding SMA to newborn screening programs [7]. However, there is a need for monitoring the response to treatment, including standardized tests of physical function conducted by physiotherapists. Although many children have a response to treatment and reach new milestones, the majority are not cured, and new phenotypes are evolving [8]. Based on the experience with SMA and the occurrence of new phenotypes posttreatment, a large group of patients will need multidisciplinary follow‐up in adult years. To have access to treatment and multidisciplinary care does not mean that all patients need or should use the entire toolbox. The benefits need to be weighed against the disadvantages experienced by the patients [9]. This is beyond the scope of this editorial, but is an important topic for further discussions.

SMA is just the beginning. Numerous orphan drugs are in various stages of development for other hereditary neuromuscular disorders. Nonetheless, the requirement for multidisciplinary teams is not solely linked to emerging therapies. More and more studies conclude that exercise, diet, respiratory and cardiac care, cognitive behavioral therapy, and other multidisciplinary approaches to disease management, including palliative care, might have a strong impact on disease progression and quality of life. This may be equivalent, or in some cases potentially also superior, to some of the emerging drug treatments [10]. There is also an opportunity to inflate effect sizes with combined treatment approaches of drug and rehabilitation interventions.

Hence, there is a pressing need for a multidisciplinary approach with robust health care teams, to foster current best practices and prepare for future advancements in medicine.

## AUTHOR CONTRIBUTIONS

**Kristin Ørstavik:** Conceptualization; writing – original draft; writing – review and editing. **Andreas Dybesland Rosenberger:** Writing – review and editing; writing – original draft. **Hanne Ludt Fossmo:** Writing – review and editing. **Marianne Nordstrøm:** Writing – review and editing; conceptualization; writing – original draft. **Marianne de Visser:** Conceptualization; writing – review and editing.

## CONFLICT OF INTEREST STATEMENT

None of the authors has any conflict of interest to disclose.

## Data Availability

Data sharing is not applicable to this article as no new data were created or analyzed in this study.
